# Magnetic Resonance predictors of arrhytmic events during the acute phase of hemodynamically stable myocarditis

**DOI:** 10.1186/1532-429X-16-S1-P263

**Published:** 2014-01-16

**Authors:** Lorenzo Monti, Lucia Occhi, Claudio Moro, Giuseppe Iacuitti, Veronica Lisignoli, Barbara Nardi, Maddalena Lettino, Luca Balzarini

**Affiliations:** 1 Radiology, Humanitas Research Hospital, Rozzano(MI), Italy; 2 Cardiology, Humanitas Research Hospital, Rozzano, Italy; 3 Cardiology, P.O. Desio, Desio, Italy

## Background

Acute myocarditis may be complicated both during acute phase and follow-up by ventricular arrhythmias or A-V block. Late Gadolinium Enhancement (LGE) extension predicts events in the follow-up, but no data are available on predictors of events during the acute phase of myocarditis in hemodynamically stable patients. We serched for imaging predictors of arrhythmic events in patients with hemodynamically stable acute myocarditis.

## Methods

Methods and Materials: 103 patients (pt) with hemodynamically stable acute myocarditis. All of them were hospitalized from the E.R. in one of 3 participating centres. Acute myocarditis was diagnosed with MR in presence of both T2 STIR edema and LGE. Clinical, bioclinical, ECG and imaging data were reviewed by a panel consensus of 1 cardiologist and 1 radiologist to adjudicate events.

## Results

80 males (77,67%) and 23 females (22,33%), 95% in NYHA class I. Mean EF =58,6% ± 10. During hospitalization in 14 pz (13,6%) were observed arrhythmic events including 1 torsades de pointes, 4 NSVT, and 1 SVT. A reduced LVEF (i.e.: below normal reference range for cardiac MR) was significantly associated with events, with OR = 8,4. Neither WBC count (OR 1,14), troponin peak (OR = 1,18), end-diastolic volume ( OR = 1,0), Late Gadolinium Enhancement mass(OR = 1,06) or T2 STIR edema mass (OR = 1,2) were related to arrhythmic events during the acute phase of myocarditis.

## Conclusions

In hemodynamically stable patients hospitalized for acute myocarditis, clinical events are mainly arrhythmic with a prevalence of 13,5%. A reduced LVEF, evaluated with MR, is the only parameter associated with clinical events during the acute phase of the disease.

## Funding

None.

